# On the thermodynamically stable amorphous phase of polymer-derived silicon oxycarbide

**DOI:** 10.1038/srep14550

**Published:** 2015-09-30

**Authors:** Liping Yu, Rishi Raj

**Affiliations:** 1 University of Colorado at Boulder Boulder, Colorado 80309, USA

## Abstract

A model for the thermodynamic stability of amorphous silicon oxycarbide (SiCO) is presented. It builds upon the reasonably accepted model of SiCO which is conceived as a nanodomain network of graphene. The domains are expected to be filled with SiO_2_ molecules, while the interface with graphene is visualized to contain mixed bonds described as Si bonded to C as well as to O atoms. Normally these SiCO compositions would be expected to crystallize. Instead, calorimetric measurements have shown that the amorphous phase is thermodynamically stable. In this article we employ first-principles calculations to estimate how the interfacial energy of the graphene networks is favorably influenced by having mixed bonds attached to them. We analyze the ways in which this reduction in interfacial energy can stabilize the amorphous phase. The approach highlights how density functional theory computations can be combined with the classical analysis of phase transformations to explain the behavior of a complex material. In addition we discover a two-dimensional lattice structure, with the composition Si_2_C_4_O_3_ that is constructed from a single layer of graphene congruent with silicon and oxygen bonds on either side.

The interaction between carbon and amorphous silica has been of long-standing interest to scientists from different communities. In the early 1950s attempts were made to introduce carbon into SiO_2_ for the purpose of enhancing the thermal and electrical conductivity of the silica glass^1^. These efforts, which entailed stirring graphite into molten silica, did not succeed because carbon is essentially insoluble in silica.

However, more recently, it has been shown that large mole fractions of carbon can be incorporated into compounds of silicon, carbon and oxygen if they are synthesized via the chemical route^2^. For example when methyltriethylsiloxane, where silicon is coordinated with three oxygen atoms and one methyl (-CH_3_) group^3^, is heated to 800–1000 °C in an inert atmosphere, then the methyl group dissociates into hydrogen, which is released to atmosphere, while carbon, bonded to silicon, along with the three O-Si bonds, are retained in the solid state, yielding a compound with an overall Si:C:O composition of approximately 1:1:3. These compounds are amorphous when examined by Bragg diffraction.

The carbon content in these materials, processed from the polymer route, can be varied over a wide range by changing the composition of the organic precursors^4^. They have come to be known, in general, as polymer-derived ceramics or PDCs. Until recently SiCO materials prepared in this way were thought to be random networks of Si, C and O, which contradicted the fact that silica and carbon are practically insoluble in one another.

Insights into the molecular structure of PDCs began to emerge from the following observations: (i) Although devoid of wide-angle X-ray diffraction peaks, the SiCO materials exhibit strong signals in Small Angle X-ray Scattering (SAXS) suggesting the presence of short range order^5^, (ii) Strong D and G peaks in Raman spectroscopy suggested the presence of extended states of sp^2^ carbon within the structure^6^, (iii) NMR measurements of ^13^C confirmed the presence of sp^2^ carbon, but, in addition, the ^29^Si spectra showed the presence of mixed bonds of SiC_4-n_O_n_ for n = 1, 2 and 3^7^, and (iv) Measurements of creep at temperatures above 1300 °C showed the remarkable result that the materials exhibited viscoelastic behavior, but not viscous flow as occurs in silica glass^8^. These observations, taken together, led to a nanodomain model for the molecular structure which is shown in Fig. 1(a)^9^. The model shows three-dimensional network of graphene sheets forming a network of nanodomains that contain within them silicon-oxygen molecules. The mixed bonds between Si, C and O lie at the interface with the graphene walls. In the model, the size of the nanodomains is calculated to be 1–5 nm, which agrees with the SAXS measurements^9^. The model not only explains the SAXS, Raman and NMR data but is also consistent with the creep resistance at high temperatures. Viscous flow is prevented by the graphene network bearing the applied load as the silica molecules within the domains relax; as such the material shows viscoelastic behavior but not steady state flow.

The validity of the graphene network model has been confirmed by molecular dynamics calculations^10^, as well as by first principles DFT calculations^11^. One such result is given in Fig. 1(b). It shows the segregation of carbon into graphene, and the formation of mixed bonds between it and silicon-based molecules. Other descriptions^12^,^13^ of the segregation of carbon in SiCO are essentially consistent with^9^; however only the latter work presents quantitative predictions of the domain size (as a function of the carbon content) which compare favorably with the measurements by SAXS.

The most remarkable recent development has been measurements of the enthalpy of formation of SiCO compounds by high temperature calorimetry which asserts that the amorphous state of these materials has a lower enthalpy than the crystalline state^14^. We seek to explain the stability of the amorphous phase of silica in the nanodomain model of Fig. 1(a), in terms of the energy of the graphene-SiO_2_ interface, which is calculated from first principles.

## Framework for analyzing the thermodynamic stability of the amorphous phase

**Figure 1:**
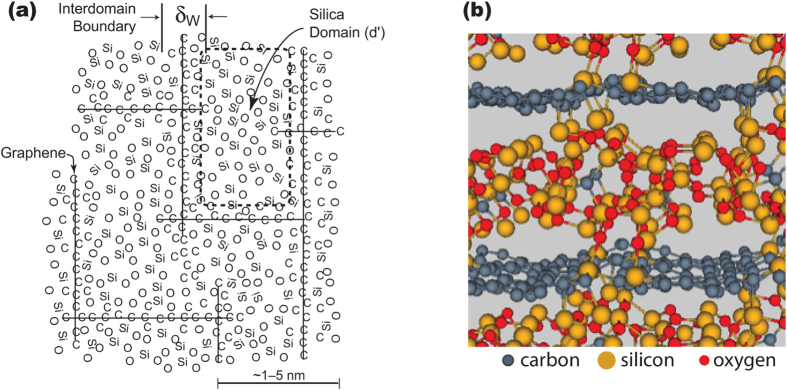
(**a**) A nanodomain model^9^ for the structure of silicon-oxycarbide. It has three features: a network of graphene that forms a cellular network, silica tetrahedra sequestered wthin the domains, and mixed bonds of Si-C-O that decorate the graphene surfaces. (**b**) First principles computer simulation of the Si, C, O compound shows the separation of graphene from SiO2. Note the presence of bridging mixed-bonds between silicon, carbon and oxygen. Courtesy: Yong-Hyun Kim, Graduate School of Nanoscience and Technology, KAIST, Korea South.

We seek to determine whether or not the amorphous phase of silica in the nanodomain model shown in Fig. 1(a) can be stabilized by the interfacial energy that it forms with graphene, via the mixed bonds between carbon, silicon and oxygen. If the amorphous phase were to crystallize then these mixed bonds will not survive, effectively increasing the interfacial energy in the nanodomain structure. If the domain size is very small, with a high interface to volume ratio, then the interfacial penalty would dominate the gain in the volumetric free energy, thereby stabilizing the amorphous network.

We follow the hypothesis that the mixed bonds of Si present at the graphene interface lowers its energy. These mixed bonds are silicon-centered tetrahedra consisting of Si bonded to oxygen on one side and the carbon atoms in graphene on the other. These bonds are the source of interfacial bonding and a lower interfacial energy. We estimate the change in the interfacial energy in terms of the cost of breaking C-Si bond in these mixed bond tetrahedra.

The unusual feature of the C-Si bonds is that they force the carbon atoms in graphene to change their hybridization from sp^2^ to sp^3^; thus the C-Si bond induces strain in the adjacent C-C bonds which leads to a minimum in the interfacial energy when calculated as a function of the density of Si bonds on the graphene face.

The graphene segments in the nanodomain structure would contain edges with the spatial frequency of the nanodomain size. The edges are defect sites where the mixed bond interactions will be stronger. We shall consider both the face and the edge coordinations of the C-Si-O mixed bonds at the interface. In this section a framework for this analysis is developed which can lead to a quantitative estimate of the domain size which can be stabilized in this way.

### Free Energy considerations for amorphous to crystal transformation

The argument for the thermodynamic stability of the amorphous nanodomain structure of Si_x_C_y_O_z_, as determined from high temperature calorimetric measurements^14^, centers on the functionalization of graphene surfaces and edges with mixed-bonds. Crystallization would require the mixed-bonds to be broken.

**Figure 2:**
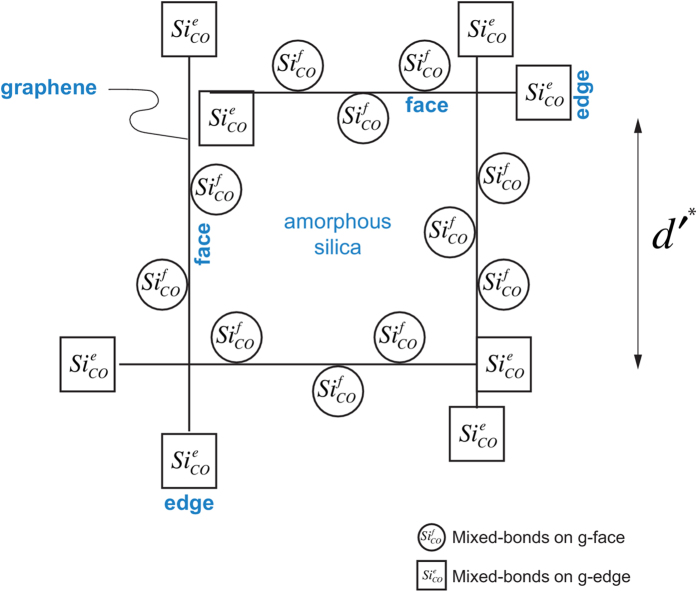
A schematic showing the mixed bonds being attached to the face atoms as well as to the edge atoms of graphene.

The mixed-bonds are attached to graphene in two ways: on the face of the graphene segments or along its edges as shown schematically in Fig. 2. We write the change in Gibbs free energy between the crystalline state (called State I) and the amorphous state (State II), by the quantity Δ*G*_*II-I*_. We assume that the mixed bonds are present in the amorphous state but broken in the crystalline state, that is, the interfacial energy of graphene, with the mixed bonds attached to it is lower than without the mixed bonds. Δ*G*_*II-I*_ is then given by
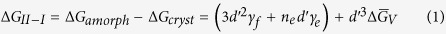
where *d*′ is the size of the nanodomains. Here 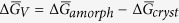 is the difference in the free energy of the amorphous and the crystalline state; it is a positive quantity since the crystalline phase has a lower free energy. The bar placed above these free energy quantities implies that they have units of energy per unit volume.

The differences in the interfacial energy of the amorphous state and the crystalline state are written as *γ*_*f*_ and *γ*_*e*_, where the subscript *f* refers to the graphene face and *e* to the graphene edges. They are equal to the energy of the bonded state where silicon forms mixed bonds with the carbon atoms in graphene, minus the energy of the separated state where the silicon-carbon bonds have been broken. Defined in this way, both are negative quantities.

The pre-factors in the interfacial energy terms, in Eq. (1), reflect the sharing of interfaces and the edge atoms between adjacent domains, which are, for simplicity, assumed to be in the shape of cubes with an edge length of *d*′. Each face of the cube is shared by two adjacent domains, thus overall three of such faces belong to one cube, hence the pre-factor of three in the *d*′^2^*γ*_*f*_ term. The other interface term refers to the edges of the graphene segments that form the cube. Here there are different possible values for the pre-factor. At one extreme it can be assumed that there is only one edge that is shared by the four cubes that meet there, in which case each edge would be shared between four cubes; since there are twelve edges, three of them would belong to a cube. For this case the pre-factor *n*_*e*_ = 3.

It is however, difficult to imagine the edges of the graphene from four adjacent cubes meeting to form a one line of a coherent carbon structure. It is more likely that *segments* of graphene form the faces of the cubes, each with their own edges that do not dovetail with the edges from the orthogonally aligned adjacent faces. In this instance four edges are shared by the four cubes that meet there, and the total number of edges per cube becomes *n*_*e*_ = 12. Thus a pre-factor three would be the minimum value for *n*_*e*_and twelve its highest possible value. In the following analysis we compute the predictions for the model for *n*_*e*_ = 3 → 12.

The form of Eq. (1) when plotted against *d*′, exhibits a minimum. Its position is analyzed by differentiating the right hand side with respect to *d*′ and setting the result equal to zero. The solution to this equation gives the value for *d*′ where the total free energy reaches its minimum value. We call it *d*′^*^.

Differentiating the right hand side of Eq. (1) gives
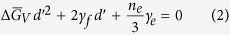


This quadratic equation has two solutions. One of them, which is necessarily negative, is not admissible. The viable solution for Eq. (2) gives the following value for the position of the minimum in the free energy
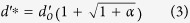
where,
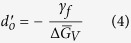
and,
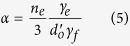
Note that in Eq. (4) 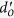
is a positive quantity since *γ*_*f*_ < 0. The quantity in Eq. (5), *α*, is positive since both *γ*_*e*_ and *γ*_*f*_ are negative quantities.

A physical interpretation of the right hand side of Eq. (3) is gained by recognizing that 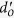, given by Eq. (4), depends only on the interfacial energy of graphene faces, while *α* recognizes the influence of edge energy. If the edge energy goes to zero, that is, there are no mixed bonds present at the edges, then *α* → 0, and 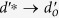. Thus *α* represents the contribution of the edge mixed bonds to the calculation of the domain size where the free energy. Note that a larger domain size implies that crystallization becomes more difficult, or, in other words, the interfacial energy opposition to crystallization grows stronger.

We now normalize the variables in Eq. (1) into non-dimensional forms. The left hand side of Eq. (1) is the total change in energy. We normalize it with respect to the product 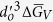, which has units of energy, since 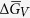
has units of energy per unit volume. Dividing both sides by this product, and substituting for 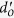 from Eq. (4) and for *α* from Eq. (5) into the terms on the right hand side of Eq. (1) leads to
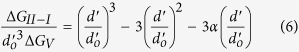


**Figure 3:**
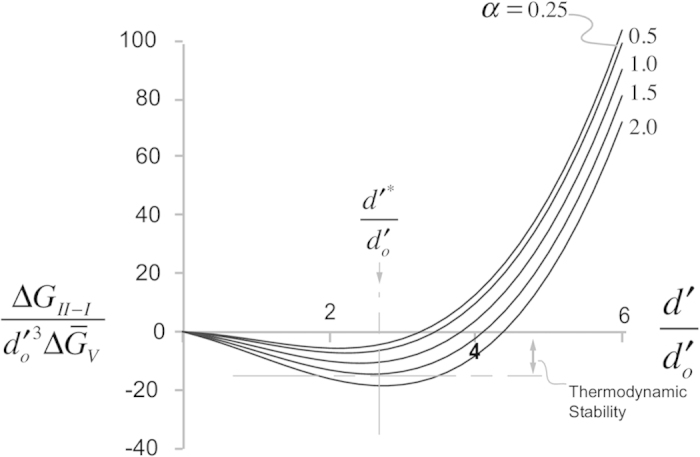
Plots of Eq. (6) showing the domain size where the amorphous state is stable. A deeper minimum and a shift of the minimum to the right implies greater stability of the amorphous state.

Plots of Eq. (6) for different values of *α* = 0.25 to 2.0 are shown in Fig. 3. The minimums become deeper and move towards a large size of the nanodomains as *α* increases, reflecting the increasing role of the edge bonds. The values for *α* are derived from first principles calculations, as described in the next section.

### The significance of mixed interface bonds at graphene edges

In order to determine the relative significance of the mixed bonds formed at the graphene face and the graphene edges, we consider the case where the only contribution to interfacial energy is provided by the mixed bonds present at the edges, without any mixed bonds being present on the face atoms.

For this purpose we omit the face energy term from Eq. (1). Retaining only the edge term gives
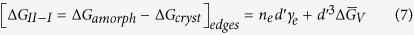


Differentiating and equating to zero gives the following result for the domain size when it is stabilized solely the interfacial energy of the graphene edges
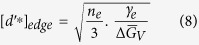


### Relating bond-energies to interfacial energies

In the next section we compute the magnitude of the binding energy of the mixed bonds to the face atoms, and to the edges of graphene. The interfacial energy depends not only on the energy per bond, but also on the concentration of the Si-C bonds, defined as the number of Si-C bonds as a fraction of the total number of carbon bonds on the surface. For example if one out of ten carbon atoms on the surface contains a Si-bond then the concentration is given by the Si/C ratio is equal to 0.1. Since the binding energy of one mixed-bond is distributed over several carbon atoms, the gain in energy per carbon atom is equal to the product of the binding energy of one bond times the concentration of the bonds. For example if the binding energy of one Si-bond is a quantity X then the binding energy per carbon atom, for a Si/C ratio of 0.1, would be 0.1X. We call this latter quantity 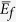 for the graphene face and 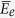 for the edges.

The interfacial parameters are related to 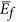 and 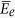 in the following way. The average interatomic spacing of carbon atoms in graphene, *α*, may be set equal to *a*^3^ = Ω, where Ω is the effective volume of carbon atom in graphite. Then the following relationships apply
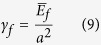
and,
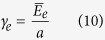


The effective volume per carbon atom is Ω = 0.0095 nm^3^, assuming the density of graphite to be equal to 2.09 g cm^–3^, gives *a* = 0.21 nm. Note that *γ*_*f*_ being face energy has units of energy per unit area while *γ*_*e*_ has units of energy per unit length.

## First principles computations

### The method

The first-principles calculations were performed using density functional theory (DFT) and plane-wave projector augmented-wave method^15^ with the exchange-correlation of Perdew-Burke-Ernzerhof^16^ as implemented in the VASP code^17^. An energy cutoff of 400 eV was used. The atomic forces were relaxed to be less than 0.03 eV Å^−1^.

The binding energy of a Si bonded to the graphene surface as a function Si-C bond concentration was calculated by using supercell approximation. The supercell consists of 60-atom graphene with 1–16 SiH_3_ molecules or 1–8 Si(OH)_3_ molecules attached to it. The graphene sheets were separated by 20 Å vacuum in the supercell. For the edges both armchair and zigzag configurations were considered.

### A two-dimensional lattice between graphene and silicon dioxide

**Figure 4:**
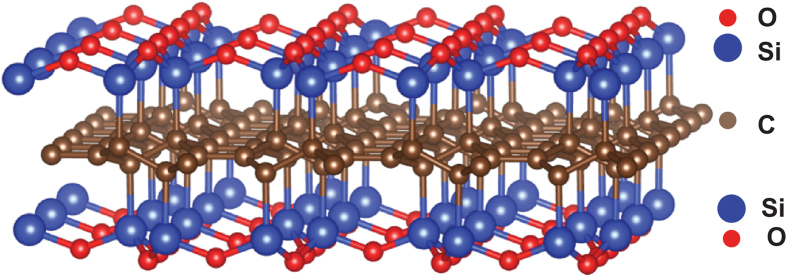
A 2-D crystal of monolayers of silicon on graphene with a binding energy of −1.2 eV per silicon atom. The stoichiometry of this structure is 2:4:3::Si:C:O.

When exploring the various geometries for attaching Si-O molecules to graphene surfaces, the periodic structure, shown in Fig. 4, was discovered. The average composition of this 2-D structure is Si_2_C_4_O_3_, that is there are two carbon atoms and 1.5 oxygen atoms for every silicon atom. The mixed bond molecules, per silicon atom, have the composition SiC_1/4_O_3/2_, with the silicon being coordinated to one carbon and three oxygen atoms. Thus one half of all carbon atoms change their electronic structure from sp^2^ to sp^3^ in order to form a bond with silicon. The binding energy per Si bond is calculated to be −1.2 eV. Therefore the quantity 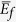 for this structure, according to the definition in the previous section, is equal to −0.6 eV since the concentration of mixed bonds 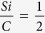. Substituting this value into Eq. (9) gives a value of 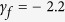 J m^–2^. This would be the work required per unit area to separate the Si-O bonds from the graphene (the separated state is the zero energy state). This magnitude of this interfacial energy is very high (for example the energy of grain boundaries in ceramics, which is also the work of fracture for separating the boundary, are typically about 1 J m^–2^). The implication is the the 2-D structure shown in Fig. 4 is energetically highly favored.

The question arises whether it is possible to synthesize this 2D lattice. The chemical route to the synthesis of silicon-oxycarbide is likely to yield a distribution of different kinds of interface structures, some of whom may indeed have the arrangement shown in Fig. 4. Calculations for the band gap for this structure gives a value of ~2.5 eV. Optical measurements of the absorption edge^18^ indeed give similar values for the band gap in silicon-oxycarbide and related materials.

### Binding energy of Si-based molecules to face atoms of graphene

We have conducted approximate calculations for the binding energy of mixed bonds of Si to graphene for the case where Si is bonded to one carbon and three oxygen atoms. The highly covalent nature of the Si-C bond and the singular sp^3^ nature of the Si orbitals is expected to yield reasonable values for the bonding energy of the Si-C bond when the valency of the remaing three orbitals is satisfied by connecting them to OH or to -H atoms. The energy of the Si-C bond is calculated for both –OH and –H configurations, and is found to be essentially similar for both of them, providing evidence for the local nature of the Si-C bond.

**Figure 5:**
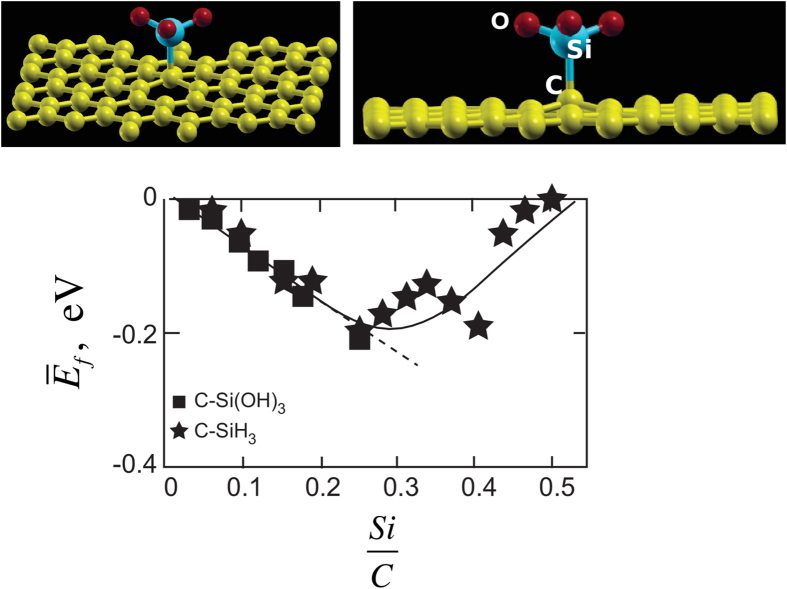
The Si-C bond causes an out-of-plane distortion of the carbon atoms. The magnitude of the binding energy varies with the concentration of the Si/C bonds, at first increasing linearly but then declining when the strain energy in the adjacent carbon atoms begins to overlap.

The calculated change in 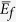
with the concentration of Si bonds, 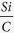, is shown in Fig. 5. Note that at first 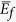
declines linearly with concentration, but only up to 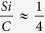. Then it begins to rise, reaching nearly zero when there is one silicon for every two carbon atoms. As shown in the molecular diagram, the sp^3^ Si-C bond pulls the carbon atoms upwards from the graphene plane, thereby inducing strain in the neighboring carbon atoms. The strain extends to the first nearest carbon. Thus if two silicon bonds were to be brought closer than the spacing between two nearest neighbor carbon atoms then the strain fields will overlap, thereby placing a penalty on the binding energy of the Si molecule to the graphene layer. (Note that the elastic strain energy is proportional to the square of the strain. Therefore the sum of the square of two equal quantities of strain is only one half of the square of twice that quantity of strain.) In summary, the energy does indeed decline linearly up to the point that silicon atoms are spaced two carbon atoms apart, but then rises when the strain fields begin to overlap.

The magnitude of 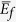 reaches a minimum value of −0.2 eV at 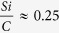. Substituting this value in Eq. (9), and recognizing that *a* = 0.21 nm, gives that *γ*_*f*_ = −0.76 J m^–2^. As a comparison the typical value for the grain boundaries in ceramics is ~1 J m^–2^. The grain boundary energy is defined as the work that would be required to separate the boundary to free surfaces. A similar definition is used for *γ*_*f*_, that is, it is the work needed to break the silicon-carbon bond to create two free surfaces.

In the above discussion we have considered mixed bonds of the SiC_1/4_O_3/2_ chemistry, that is, where Si is coordinated to one carbon (in the graphene) and three oxygen atoms. Attempts to model a situation where one silicon is bonded to two carbon atoms on the graphene face were unsuccessful, yielding very weak bonds. However, strong interaction was obtained if a carbon atom in the graphene face was removed creating a carbon-vacancy, and the Si was inserted into the vacancy to form three bonds with carbon, with the one Si-O bond lying normal to the graphene plane. In this instance a very high value of the binding energy (−3.14 eV) between the Si and graphene was obtained. If these bonds do indeed form while the polymer is converting into the ceramic then their influence on the interfacial energy, and thereby on the stability of the amorphous phase is likely to be profound.

In the following section we analyze the bonding of Si-O molecules to carbon atoms placed at graphene edges. The edge energy, *γ*_*e*_ is a line energy, and would be equal to the work required to separate the interface (please see Fig. 5) along the edge of graphene.

### Binding energy of Si-based molecules to the edge atoms of graphene

**Figure 6:**
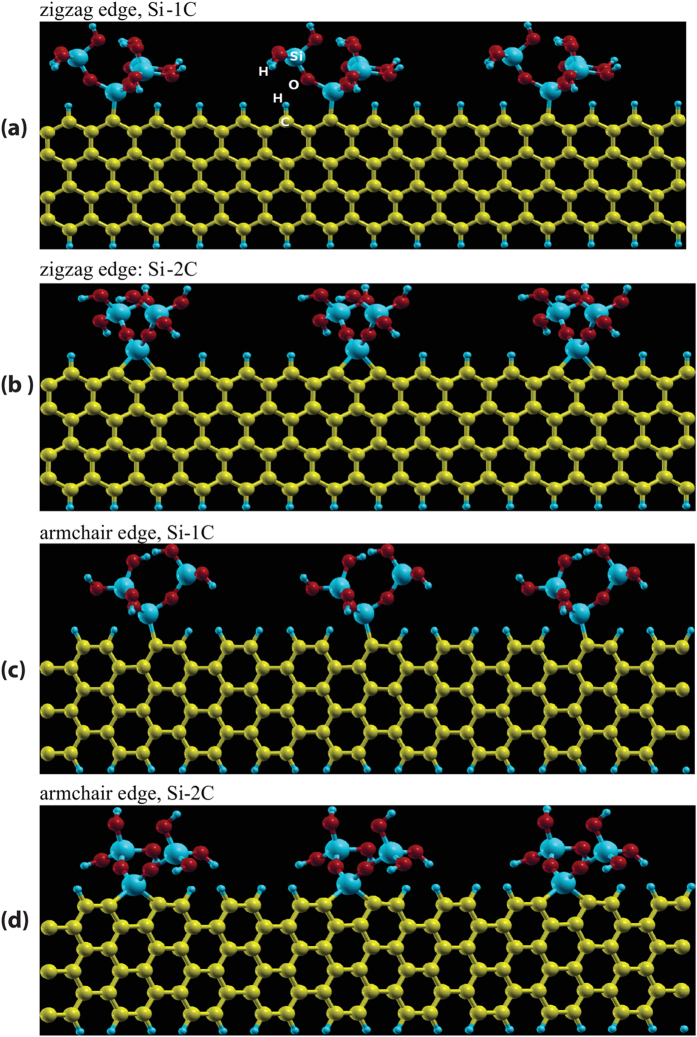
Atomic structure of mixed-bonds placed at the zigzag and armchair edges of graphene. The outermost oxygen atoms, shown in the red color, are capped with hydrogen atoms (small spheres in blue). Si and C atoms are shown in the blue and yellow color respectively.

The edges of graphene consist of dangling bonds. Therefore the intrinsic energy of the edges is much higher than of graphene surfaces. The Si mixed bonds attached to the carbon atoms at these edges can produce a significant reduction in energy. Results from these simulations where Si atoms bond to one or to two carbon atoms at edges of either zigzag or armchair configurations are shown in Fig. 6. The energy associated with these configurations are summarized in Table. 1.

There is no perceptible strain associated with the Si bonds attached to the edges, which stands in contrast to the Si atoms bonded to the face atoms of graphene. Therefore the (magnitude of the) interfacial energy is likely to rise linearly with the number concentration of Si atoms present at the edges. However, visual inspection of the molecular configurations suggests that steric hindrance will prevent the Si atoms being closer than approximately 4 carbon atom-spacings for the single Si-C bonds, and 8 carbon atom-spacings for the double Si-C bonds. Substituting the calculated values for the binding energy of the Si mixed-bonds into the relationship for 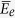 and *γ*_*e*_, from Eq. (10), gives the values for the edge interfacial energy. The values for *γ*_*e*_ obtained in this way for the four geometries of the graphene edges are listed in Table 1. They range from 0.54 × 10^–10^ J m^–1^ to 0.77 × 10^–10^ J m^–1^, with the lowest value corresponding to the Si-1C, zig-zag configuration and the highest to the Si-2C, zig-zag configuration, as given in Fig. 6. Here 1C and 2C implies that the Si is bonded to one or two carbons at the graphene edge.

### Comparison with experiments 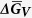


It is possible to seek a comparison between the measurements of the average size of the nanodomains in the amorphous structure of SiCO, measured by SAXS, which have been reported in ref. 9, and their value predicted from above analysis. The prediction depends on two quantities, the difference in the Gibbs Free energy between the crystalline and the amorphous states, 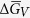, and the interfacial energies.

The high temperature calorimetric measurements^14^ provide the difference between the enthalpy of the amorphous and the crystalline state, that is, 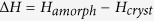. The free energy is given by 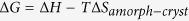. Since the amorphous phase has a higher entropy than the crystalline phase 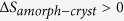, and therefore 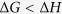. Thus the free energy 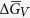 would be somewhat less than the difference in enthalpy. In the present analysis we are overestimating the free energy by assuming that it is equal to the enthalpy measured in the calorimetry experiments^14^.

Below we estimate the size of the nanodomains assuming that the free energy is equal to the enthalpy measured from calorimetric experiments. It is immediately evident from Eq. (4) that an overestimate of the free energy would lead to an underestimate of the size of the nanodomains. Thus the actual values of the domain size are likely to be greater than the calculated values, given below, by the same fraction that we are overestimating the free energy.

**Figure 7:**
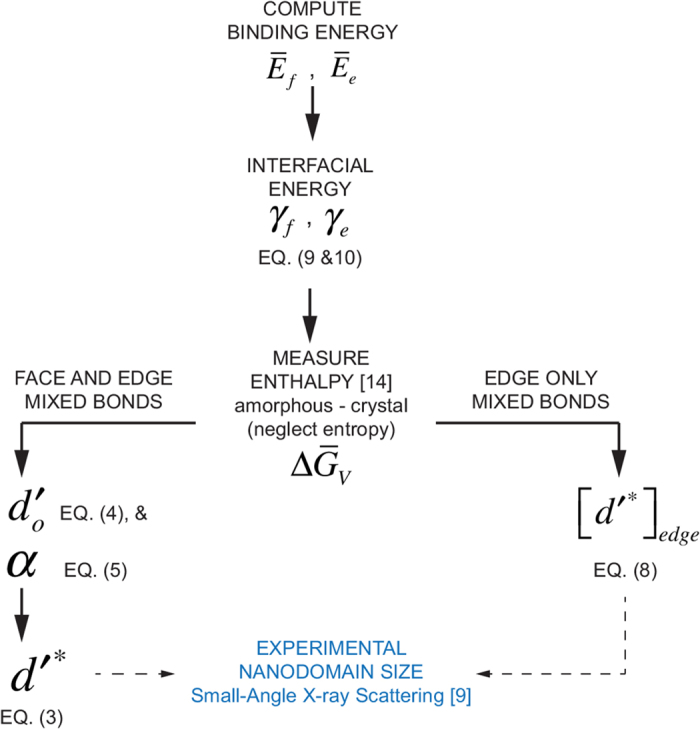
The methodology for combining first principles computations with closed form models to analyze the behavior of a complex material, in this instance the amorphous state of polymer-derived SiCO constructed from nanodomains created by networks of graphene.

The procedure and the equations that lead to the comparison between theory and experiment are outlined in Fig. 7. The interfacial energies, *γ*_*f*_ and *γ*_*e*_ are obtained from the computed values for 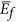 and 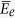 using Eq. (9) and (10). We use the lowest (the most negative) values of *γ*_*f*_ and *γ*_*e*_ for comparison with experimental data. The lowest value for *γ*_*f*_, as calculated above is −0.76 J m^–2^, and the lowest value for *γ*_*e*_ is −0.77 × 10^–10^ J m^–1^.

The enthalpy of the amorphous state relative to the crystalline state was reported in ref. 14 in units of kJ mol^–1^. It was converted to 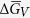, which has units of J m^–3^, by calculating the molar volume of Si_x_C_y_O_z_ from its composition and assuming a density of 2 g cm^–3^ (the values for the density of SiCO range from 2.0 to 2.2 g cm^–3^ 19).

The values for *γ*_*f*_, *γ*_*e*_, and 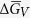 may now be inserted into Eq. (4) to calculate 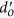, from Eq. (4), and *α* from Eq. (5). These values can now be used to obtain the theoretical estimate of the domain size, *d*′^*^, from Eq. (3). In this case the interfacial energy includes the mixed bonds at the faces and the edges of graphene.

The prediction for the domain size by considering only the bonds at the edges, and ignoring the bonds on the faces of the graphene segments, is given by Eq. (8).

As had been discussed earlier the number of graphene edges per cube, *n*_*e*_, can range from 3 to 12. Thus we consider these two limiting values for estimating the domain size. The larger value of *n*_*e*_ would be expected to lower the interfacial energy further thereby rendering greater stability to the amorphous phase, which would then translate in to a larger value for the domain size.

The high temperature calorimetry data^14^ for five compositions of Si_x_C_y_O_z_, written as S1-S5, are summarized in Table 2. The values for 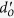 are given in a column on the right hand side. Note that they range from 0.37 to 0.96 nm. The value 0.05 nm for S5 is an outlier, principally because the free energy measurement for this composition was abnormally high. Recall from Eq. (3) that the domain size is predicted to be equal to 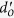 when *α* = 0, that is in the absence of edge bonds. The experimental values of the domain size that usually lie in the 1–3 nm range, are significantly larger than estimated from 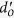, implying that the edge mixed-bonds indeed make a significant contribution to the stability of the amorphous phase.

The results from the calculation that includes both the face and the edge mixed-bonds are given in Table 3. The theoretical predictions for *n*_*e*_ = 3 and *n*_*e*_ = 12 are given by *d*′^*^ in the second column. The theoretical value for the domain size which considers only the edge bonds, [*d*′^*^]_*edge*_, are given in the third column. The right hand column gives the experimental values of the domain size four the specimens S2–S5, as measured by small angle scattering and reported in ref. 9. Disregarding S5, which appears to be an outlier because of its anomalously large value for 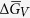, the agreement between theory and experiment is remarkably good.

In general, the best agreement is obtained by considering the interface energy to be influenced by both the face and the edge bonds of graphene. Considering only the edge bonds, without any face bonds, under estimates the domain size. Considering only the face bonds, without the edge bonds, also under estimates the domain size; these results are given by 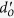 in Table 2. *We believe this to be an important finding: that both face and edge mixed-bonds play a role in the stability of the nanodomain structure of SiCO.*

## Discussion

We have employed a synergistic approach to understand and predict the stability of the amorphous nature of the nanodomain structure of polymer derived SiCO. The framework for the analysis draws from classical modeling approaches in materials science, while the quantitative information regarding the structure and the energetics of the interface is obtained from first principles calculations, gives heft and predictability to the overall analysis, enabling a quantitative comparison between theory and experiment. In this way, a seemingly complex problem can be approached to obtain meaningful results. *Neither the classical analysis, nor the computational results, on their own could have achieved this objective.*

It is, however, important to recognize that the question addressed by the present work arose out of diverse kinds of experimental data, a model that captured these findings into a molecular framework^9^, and thermodynamic measurements that not only proved the unusual stability of the amorphous structure but also provided quantitative measurements for the difference between the enthalpy of the crystalline and amorphous states^14^.

The concept that drove the present analysis was that the size of the nanodomains could be stabilized by the interfacial bonding between silica and graphene, promoted by mixed bonds between carbon, silicon and oxygen. We postulated that this interface would have to separate by breaking the carbon-silicon bonds if the structure were to crystallize, thus opposing crystallization. A larger interface area (implying a smaller domain size) and a higher (more negative) interfacial energy between silica and carbon would therefore promote the stability of the amorphous phase. The credibility of this concept could be established only by quantitative analysis. The estimates of the interfacial energy from first principles calculation and the measurements of the free energy of crystallization could now be molded into a model to compare theory with experiment. The results in Table 3, which give remarkably good agreement between the prediction and the measurement of the domain size, demonstrate the success of the approach.

The exercise of comparing theory with experiment reveals new insights into the importance of the interface structure. The bonds between silicon and carbon can occur in two configurations: at the faces, and at the edges of the graphene segments. The results in Table 2 show that both 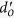, which considers only the face bonds, or [*d*′^*^]_*edge*_, which considers only the edge bonds, underestimate the experimental values of the domain size. Clearly both faces and edges play a significant role in the stabilization of the nanodomain structure; this fact had not been recognized in the original model^9^.

The computations have revealed interesting new scenarios for the graphene-silica interface structure and energetics. A most remarkable prediction is of a self-standing two-dimensional lattice, which given in Fig. 4. Here, one graphene layer is congruent with a specific arrangement of silicon and oxygen atoms on either side. The composition of this 2-D lattice is Si_2_C_4_O_3_, and its interfacial energy is calculated to be −2.1 J m^–2^; this would be the work required to separate the graphene from the silicon-oxygen layers. It is intriguing to consider if such stand-alone 2-D structures can be synthesized from the chemical route. They probably exist, sporadically, in the conventionally processed polymer derived ceramics; for example the band gap estimated for this structure, 2.5 eV, agrees well with the experimental measurement of the absorption edge^18^.

The computational results raise further questions about the C-Si-O coordination of the mixed bonds that form the interface. Here we have considered Si bonded to one carbon on the graphene faces with the remaining three bonds being occupied by oxygen. Attempts to connect Si to two carbon atoms on the graphene surface and then to two oxygen atoms yielded very weak bonding. However, if the a Si was inserted to fill a defect site where a carbon atom in the graphene was absent, thereby bonding to three carbon atoms and then to one oxygen atoms then a binding energy of −3.21 eV was achieved. Therefore the genesis of how Si mixed-bonds may become incorporated into defect sites in graphene as the nanodomain structure evolves from the polymer is an intriguing question. Certainly the edges of graphene present ready-made defect sites where the mixed-bonds can attach, and we have considered this issue in detail in the present analysis.

An interesting feature of the face-bonds is dependence of the interfacial energy on the density of the Si bonds on the surface. The energy is lowest for silicon-to-carbon ratio of 0.25. At lower concentrations the energy is weaker because there are fewer bonds. However, at higher concentrations the strain induced in the graphene when the Si atom attaches to a carbon atom, forcing it to change from sp^2^ to sp^3^ hybridization, has a decay length of about one carbon spacing. The overlap of the strains from adjacent Si atoms then causes the overall binding energy to weaken.

The above result, which is described quantitatively in Fig. 5, has implications in the composition regime where the amorphous structure may stable. It implies that the most likely region where the amorphous structure can be stable would be midway between the SiC-SiO_2_ tie line and the carbon apex, which is generally held to be correct. It has been difficult to fabricate glassy PDCs close to the tie-line and also close to the carbon rich portion of the composition diagram.

Finally, we discuss the approximations that have been used to pose the problems in a way that has made the present first principles computations feasible. We have assumed the Si-molecules to be terminated in –OH or –H bonds on the other side of graphene. Strictly speaking they would be a part of the SiO_2_ network within the interior of the domains. This approximation raises two issues: how such a configuration affects the strength of the Si-C bonds, and what may be the effect of the constraint from the silica network on the Si-C bond. The energy of Si-C bond for –OH and –H configurations are essentially the same in Fig. 5, suggesting that the electronic structure of the Si-C bond is local, and therefore, the approximation is probably acceptable. The strain that would be induced on the Si-C bond by an extended silica network is more difficult to estimate. Normally the binding energy corresponds to about 20–25% of elastic strain. Thus, if the constraint induces a strain of 2%, then the error in the calculated energies will be about 10%.

A fraction of the carbon atoms at the graphene edges will necessarily have dangling bonds since they cannot all be bonded to Si. In the analysis we have terminated them with hydrogen atoms. The other possibility would be that they are bonded, at an angle, to the edges of the graphene from adjacent cells. This complexity is of a high level and unlikely to be successfully modeled by density functional theory calculations. The presence of some hydrogen in the polymer-derived materials is controversial since hydrogen is released over 700 °C to 1250 °C during processing. Therefore residual hydrogen could have a role in stabilizing the nanodomain structure of the amorphous state.

The high temperature calorimetry measures the enthalpy of formation, while the thermodynamic stability is related to the Gibbs free energy which includes entropy. Since the entropy of the amorphous state would be greater, the difference in the free energy of the amorphous and the crystalline states would be smaller (entropy lowers the free energy) than estimated here. It also means that the actual values for 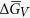 may be smaller than those we have used in the present analysis. A smaller value for 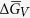 will encourage stability of the amorphous phase, which will lead to slightly larger values for the domain size.

## Summary

First principles computations have been combined with classical analysis of phase stability to gain quantitative insights into the stability of polymer derived SiCO. The question of the role of the interfacial energy between graphene and silica in stabilizing the amorphous nature of the nanodomain structure of these materials has forged a confluence between these two approaches. The interfacial energy is calculated from specific configurations of mixed bonds of C-Si-O that create bonding between graphene and silica. Mixed bonds at the faces and at the edges of the graphene segments are considered. It emerges that both types of bonds make meaningful contribution to the interfacial energy. Only when both are considered do we obtain a satisfactory agreement between the measured and predicted values of the size of the nanodomains.

The computations have revealed a new two-dimensional lattice of a middle layer of graphene bonded to Si-O layers on either side. This structure has a 2:4:3 (Si:C:O) stoichiometry, which lies close to the middle of the Si-C-O composition diagram. It is energetically highly favored relative to the other cases of interfacial energies that we have considered here. It is intriguing to think if this 2-D lattice can be fabricated from the chemical route and what kinds of new functional properties it may possess.

We find that the energy of the graphene-silica interface has its lowest, or the most favored value midway between the SiC-SiO_2_ tie-line and the C-apex. Thus it is anticipated that the amorphous nanodomain structure is likely to be most stable in the middle regime of the Si-C-O composition diagram.

## Additional Information

**How to cite this article**: Yu, L. and Raj, R. On the thermodynamically stable amorphous phase of polymer-derived silicon oxycarbide. *Sci. Rep.* **5**, 14550; doi: 10.1038/srep14550 (2015).

## Figures and Tables

**Table 1:** Binding energy for the Si mixed bonds to the zigzag and armchair edge structures of graphene, where Si bonds to either one or to two carbon atoms at the edge.

	*Si bonded to one C*	*Si bonded to two C*
	eV	eV
zigzag	−4.04	−5.64
armchair	−3.78	−6.26
	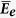	
	*(Si/C)* = *1/4*	*(Si/C)* = *1/8*
zigzag	−1.01	−0.71
armchair	−0.95	−0.78
	*γ*_*e*_ Eq. (10)	
	**J/m**	**J/m**
zigzag	−7.70E-10 (a)*	−5.38E-10 (b)
armchair	−7.21E-10 (c)	−5.97E-10 (d)

*(a), (b), (c) and (d) designations refer to Fig. 6.

**Table 2:** The values for the parameters used in the estimate of the domain size.

Sample	Si_x_C_y_O_z_			Molar Volume(density = 2 gcm^−3^)	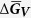 14	*γ_f_* Eq. (9)	*γ_e_* Eq. (10)	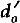 Eq. (4)	*α* Eq. (5)	
	x	y	z	m^3^mol^–1^ ; 10^–6^	J m^–3^ ; 10^9^	J m^–2^	J m^–1^ ; 10^–10^	nm	n_e_ = 3	n_e_ = 12
S1	0.36	0.12	0.51	9.94	1.99	−0.76	−7.7	0.38	2.5	9.9
S2	0.30	0.27	0.44	9.24	0.79	−0.76	−7.7	0.96	1.0	3.9
S3	0.27	0.36	0.37	8.92	3.84	−0.76	−7.7	0.20	4.8	19.1
S4	0.28	0.44	0.28	8.78	2.03	−0.76	−7.7	0.37	2.5	10.1
S5	0.26	0.50	0.24	8.55	15.0	−0.76	−7.7	0.05	18.6	74.6

The results are given in Table 3

**Table 3:** A comparison between the estimated and measured values for the domain size.

Sample	*d*′^*^ Eq. (4)		[*d*′^*^]_*edge*_ Eq. (9)		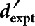 Ref. 9
	n_e_ = 3	n_e_ = 12	n_e_ = 3	n_e_ = 12	
	nm		nm		nm
S1	1.1	1.6	0.61	1.23	n/a
S2	2.3	3.1	0.97	1.95	2.3
S3	0.7	1.1	0.44	0.88	1.7
S4	1.1	1.6	0.61	1.22	1.1
S5	0.3	0.5	0.22	0.45	1.4
